# Compatibility with *Panax notoginseng* and *Rehmannia glutinosa* Alleviates the Hepatotoxicity and Nephrotoxicity of *Tripterygium wilfordii* via Modulating the Pharmacokinetics of Triptolide

**DOI:** 10.3390/ijms19010305

**Published:** 2018-01-19

**Authors:** Qichun Zhang, Yiqun Li, Mengzhu Liu, Jinao Duan, Xueping Zhou, Huaxu Zhu

**Affiliations:** 1 Jiangsu Collaborative Innovation Center of Chinese Medicinal Resources Industrialization, Nanjing University of Chinese Medicine, 138 Xianlin Rd., Nanjing 210023, China; zhangqichun@njucm.edu.cn (Q.Z.); liyiqunlyq@163.com (Y.L.); mengzhu.liu@live.cn (M.L.); dja@njucm.edu.cn (J.D.); zhouxp1960@163.com (X.Z.); 2 Department of Pharmacology, School of Pharmacy, Nanjing University of Chinese Medicine, 138 Xianlin Rd., Nanjing 210023, China; 3 Jiangsu Key Laboratory for High Technology Research of Traditional Chinese Medicine Formulae, 138 Xianlin Rd., Nanjing University of Chinese Medicine, Nanjing 210023, China

**Keywords:** *Tripterygium wilfordii*, triptolide, hepatotoxicity, nephrotoxicity, pharmacokinetics

## Abstract

*Tripterygium wilfordii* (TW) and the representative active component triptolide show positive therapeutic effect on the autoimmune disorders and simultaneously ineluctable hepatotoxicity and nephrotoxicity. Combinational application of *Panax notoginseng* (PN) and *Rehmannia glutinosa* (RG) weakens the toxicity of TW according the clinical application of traditional Chinese medicine. This article was aimed at the mechanism of decreasing toxicity of TW by the combinational application of PN and RG. Biochemical and pathohistological analysis were utilized to assess the toxicity on liver and kidney in rats administrated with TW, TW-PN, TW-RG and TW-PN-RG for 3 and 7 days. Meanwhile, the pharmacokinetics profiling of triptolide and wilforlide A was determined based on the plasma concentration analyzed by ultra-high performance liquid chromatography-tandem mass spectrometry (UPLC-MS/MS). TW-induced alkaline phosphatase (ALP), the marker for liver injury, was enhanced from 22.83 ± 1.29 to 40.73 ± 1.42 King’s unit/100 mL (*p* < 0.01) at day 7. TW-PN-RG decreased the serum ALP of TW-treated rats at 30.15 ± 1.27 King’s unit/100 mL (*p* < 0.01). For nephrotoxicity, TW pronouncedly elevated serum creatinine (SCr) in rats from 20.33 ± 1.77 to 49.82 ± 2.35 μmol/L (*p* < 0.01). However, rats treated with TW-PN-RG showed lower SCr at 30.48 ± 1.98 μmol/L (*p* < 0.01). Moreover, TW-PN-RG significantly decreased the TW-induced elevation of total bilirubin (T-BIL), alanine amino transferase (ALT), aspartate amino transferase (AST), blood urea nitrogen (Bun), and reversed the TW-resulted pathohistological characteristics of liver and kidney. The delayed time to reach C_max_ (T_max_) and reduced maximum concentration (C_max_) and area under plasma concentration-time curve (AUC) of triptolide and wilforlide A were explored in rats with combinational formulas. Synergism of PN and RG obviously prolonged the half-life (t_1/2_) and apparent volume of distribution (V_d_), but exerted no action on the clearance rate. The compatibility of TW, PN and RG influences intracorporal process of both triptolide and wilforlide A on the steps of absorption and tissue distribution contributing to less toxicity of TW on liver and kidney.

## 1. Introduction

*Tripterygium wilfordii* (TW), also known as thunder god vine, is a perennial vine-like plant from *Tripterygium* genus of Celastraceae family, which widely distributes among the south of the Yangtze River and Southwest China. TW is a routine and important medicine in traditional Chinese medicine. The properties and actions of TW are described as dispelling pathogenic wind and removing dampness, promoting blood circulation and freeing meridians, detumescence for suppressing pains, destroying parasites, and detoxifying.

Clinically, TW is employed singly or combined with other medicinal materials to therapy diseases associated with autoimmune and inflammation [1,2]. Through regulating immune disorders and inhibiting severe inflammation reaction, TW and its representative active component, triptolide, exhibit powerful and positive efficiency against rheumatoid arthritis, systemic lupus erythematosus, nephritis, experimental autoimmune encephalomyelitis, and psoriasis, all of which, presently still lack effective and reliable therapeutic measures [3,4,5,6]. Moreover, triptolide induces pro-apoptotic proteins involving caspase-3, caspase-9, and poly ADP ribose polymerase in gallbladder cancer cells [7]. Overcoming chemoresistance in prostate cancer, downregulating the nuclear factor kappa-light-chain-enhancer of activated B cells (NF-κB) signaling of pancreatic cancer and inhibiting cell viability of hepatocellular carcinoma are recently documented [8,9]. 

These intriguing and undoubted therapeutic effects of TW in both clinics and animal experiments, however, do not promote the clinical application readily due to the narrow therapeutic window, resulting in obvious side effects on the kidney, liver, reproductive system, etc., accompanied with its positive pharmacological actions [10,11,12]. Chemical investigation disclosed the complex components in TW such as diterpenoids (triptolide), triterpenoid (wilforlide A) and alkaloids (wilfordine, wilforine, and wilforgine), etc. Bioactive chemical, triptolide, is well known as the primary constitute responsible for the adverse reactions and the therapeutic effects [13]. To fight that disadvantage, three-fold measures are presently applied. One is modification of dosage form like solid lipid nanoparticles (SLN), an emergency drug delivery system, which modulates the pharmacokinetic characteristics of the bioactive constitutes, especially decreasing the maximum concentration with no or less influence on the area under curve [14]. Development of derivations of the active component is another pivotal approach, which maintains the same original nucleus structure and similar pharmacodynamics and reduces the accumulation in the liver and kidney [15]. The last measure is to combine with other chemical medicines or herbal material contributing to the expected curative effect with synergistic action [16,17]. Based on the well-designed toxic evaluation on TW or triptolide performed in in vivo or in vitro [18,19,20], pharmacological assay of these chemicals accompanied with the evaluation of toxicity was taken to explore the detail chemical background of TW in vitro.

In traditional Chinese medicine, formula is the basic prescription system, which commonly is composed of several medicinal materials. In a receipt, one is the principal medicinal materials, the others play a subsidiary role to reinforce the action of the former and/or counteract the toxicity or side effects of the former. During the treatment of diseases associated with autoimmune disorder, TW usually acts as the principal medicinal, while other combined materials are designed to neutralize the toxicity of TW according to the combinational principle of traditional Chinese medicine. To explore the rational and feasible of combination of a prevalent formula including TW, *Panax notoginseng* (PN) and *Rehmannia glutinosa* (RG) (20 g:6 g:25 g) from the Rheumatism Department of Jiangsu Province Hospital of Traditional Chinese Medicine (Nanjing, China), an investigation was designed to reveal the effect of TW on the liver and kidney and depict the characteristics of pharmacokinetics of triptolide and wilforlide A with or without PN and RG in a determined dosage.

## 2. Results

### 2.1. Method Validation

**Figure 1:**
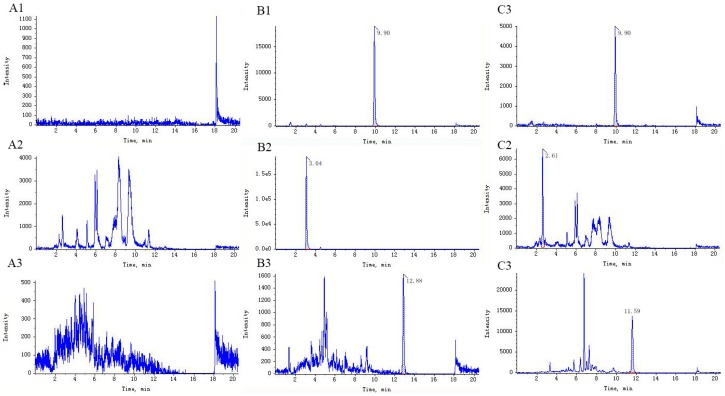
Representative chromatograms of: (**A1**–A**3**) rat blank plasma; (**B1**–**B3**) rat plasma spiked with fenofibrate, triptolide and wilforlide A; and (**C1**–**C3**) rat plasma sample after administration of *Tripterygium wilfordii* (TW).

The retention time of fenofibrate, triptolide, and wilforlide A were 9.90, 3.04, and 12.88 min, respectively. No significant endogenous peaks were observed within the time when fenofibrate, triptolide, and wilforlide A were detected. Representative ultra-high performance liquid chromatograph (UPLC) chromatograms are shown in Figure 1.

The standard calibration curve of triptolide was linear over the range 0.16–20 μg/mL with good linearity (*R*^2^ = 0.9958) and the typical equation for the calibration curve was *Y* = 1.1576*X* + 0.7451. And the standard calibration curve of wilforlide A was linear over the range 0.06–8 μg/mL with good linearity (*R*^2^ = 0.9972) and the typical equation for the calibration curve was *Y* = 0.06684*X* + 0.01024.

The analytical precision of triptolide and wilforlide A in rat plasma samples is shown in Table 1. The low concentrations of triptolide and wilforlide A are 0.63 and 0.25 μg/mL. The middle concentrations of triptolide and wilforlide A are 2.50 and 1.00 μg/mL. The high concentrations of triptolide and wilforlide A are 10.00 and 4.00 μg/mL. The method showed perfect precision within the intra-day and inter-day.

The relative standard deviation (RSD) of stability of triptolide were 7.36%, 7.85%, and 8.10% at the concentrations of 0.63, 2.50, and 10.00 μg/mL, respectively. The RSD of extraction recoveries of wilforlide A were 7.05%, 6.83%, and 7.52% at the concentrations of 0.25, 1.00, and 4.00 μg/mL, respectively.

The extraction recoveries of triptolide were 96.82%, 93.65%, and 95.14% at the concentrations of 0.63, 2.50, and 10.00 μg/mL, respectively. The extraction recoveries of wilforlide A were 96.52%, 93.85%, and 95.76% at the concentrations of 0.25, 1.00, and 4.00 μg/mL, respectively.

In summary, the validated UPLC method was reproducible and reliable to determine triptolide and wilforlide A in rat plasma samples.

### 2.2. Compatible Formulas with Panax notoginseng (PN) and/or Rehmannia glutinosa (RG) Attenuate Tripterygium wilfordii (TW)-Induced Hepatic Injury

There are significantly increased total bilirubin (T-BIL), alanine amino transferase (ALT), aspartate amino transferase (AST), and alkaline phosphatase (ALP) in rat serum after orally administrated with extract of TW for 3 and 7 days. Both PN and RG decreased pronouncedly the levels of T-BIL, ALT, AST, and ALP in rat serum induced by TW at day 3 and 7, respectively. Compared with TW-RG group, lower concentrations of the biomarker of liver function were shown in TW-PN group, which indicated the pivotal role of PN in the compatibility of the complete formula. Positive and affirmative synergism of PN and TG in neutralizing the liver toxic property of TW was observed in this study shown in Table 2.

### 2.3. Compatible Formula with PN and/or RG Lessen TW-Induced Kidney Toxic

Beside the liver injury, the toxicity of TW on kidneys is also a concern. Both blood urea nitrogen (BUN) and serum creatinine (SCr) of rat serum indicating kidney function were analyzed following the dosing of TW, TW-PN, TW-RG, and TW-PN-RG within a duration of 7 days (Table 3). A symbol of kidney dysfunction was demonstrated in the TW-treated rats having obvious enhancement of BUN and SCr. The complementary component of the formula either PN or RG shows the ability to ameliorate the abnormal of kidney injury to some normal degree. Consistent with the combinational influence of PN and RG on the side effect of TW in liver, significant synergy between PN and RG was shown to reverse the dysfunction of kidney in TW-treated rats with much lower levels of BUN and SCr.

### 2.4. Histological Assay of Liver and Kidney of Rats Administrated with TW or TW-Containing Formulas

**Figure 2:**
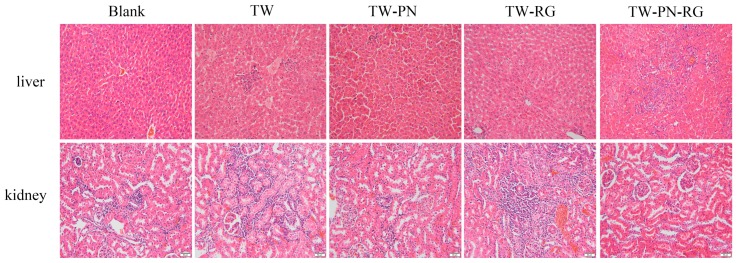
Histopathological examination of liver and kidney with hematoxylin and eosin stain (40×).

The liver and kidney tissues were stained with hematoxylin and eosin (Figure 2). Compared with the rats with saline, several mild histopathologic characteristics were observed in the liver and kidney of those with TW. In the portal area of liver, there are obvious inflammatory cell infiltration accompanied with vasocongestion and hydropic degeneration. The phenomena of liver cells necrosis and fibroplasia are also explored in these TW-treated livers. Except the slight infiltration of inflammatory cells, it’s hard to observe the aforementioned morphological properties of TW group in the liver of TW-PN and TW-PN-RG group. The present histopathologic characteristics of liver of rats with TW-RG are hydropic degeneration and inflammatory cell infiltration. In the kidney, TW resulted in moderate hydropic degeneration in renal tubular epithelium and vasocongestion and inflammatory cell infiltration in the renal interstitial. Moreover, mild vasocongestion and sclerosis were observed in the glomerulus of TW-treated rats. Minor vasocongestion and inflammatory cell infiltration in kidney are the primary histopathologic change in rats with TW-PN or TW-RG. Those preceding vasocongestion, inflammation, hydropic degeneration, and sclerosis do not appear in the kidney of rats administrated with completes formula TW-PN-RG.

### 2.5. Pharmacokinetics Process of Triptolide in Rats with TW or TW-Containing Formulas

**Figure 3:**
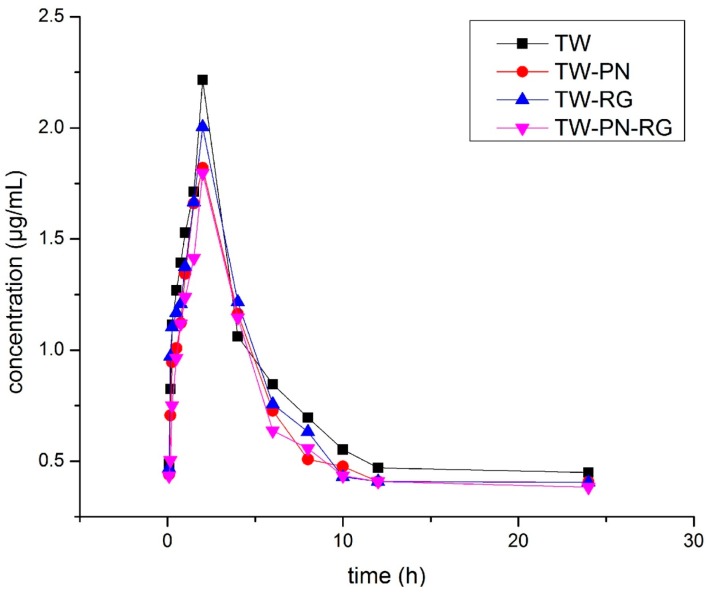
The plasma concentration-time curves of triptolide in rats.

Upon combinating with PN or PN-RG, the area under plasma concentration-time curve (AUC_(0–t)_) of triptolide was significantly decreased in the rats plasma. The compatibility of RG led to no obvious less AUC_(0–t)_ than that of TW administration along. Otherwise, all the AUC_(0–∞)_ of triptolide of TW conbinated with PN, RG or PN-RG were descendent smoothly consistent with the corresponding clearance (CL) of triptolide in rats with TW or TW-containing formulas, which indicated that PN and PN-RG primarily interfered the absorption of triptolide but not the process of metabolism and elimination. The delayed absorption of triptolide induced by PN or PN-RG resulted in extended T_max_ and declined C_max_ of triptolide. Although RG prolonged the T_max_ similarly, higher C_max_ was demonstrated than that of TW with PN or PN-RG. The combination of PN and RG (PN-RG) pronouncedly broaden the volume of distribution (V_d_) resulting in extended t_1/2_ indicated relatively expanded therapeutic duration (Figure 3 and Table 4).

### 2.6. Pharmacokinetics Process of Wilforlide A in Rats with TW or TW-Containing Formulas

**Figure 4:**
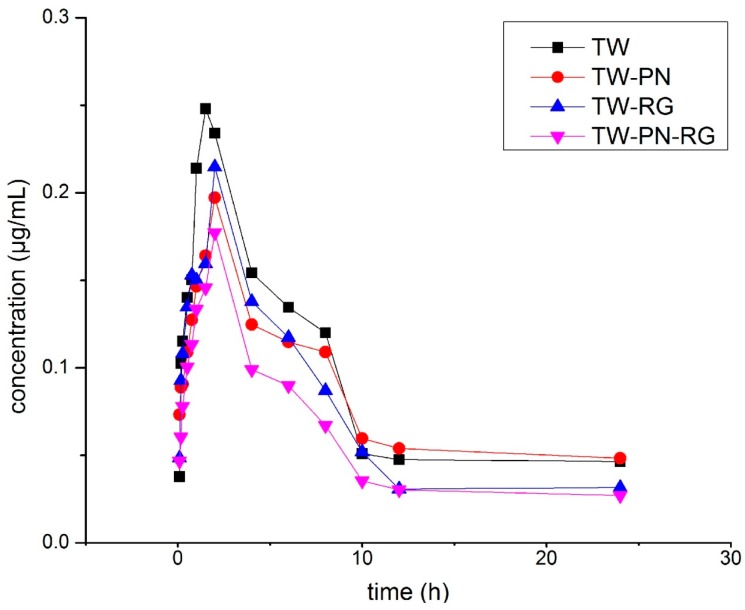
The plasma concentration-time curves of wilforlide A in rats.

The AUC_(0–∞)_ of wilforlide, another key component of TW, were decreased significantly in the rats with TW-PN, TW-RG, and TW-PN-RG compared with the those with TW singly. Dramatic reductions of AUC_(0–t)_ of wilforlide was shown in the TW-PN-RG group due to the synergism of PN and RG. All these extended t_1/2_ of wilforlide in groups of TW-PN, TW-RG and TW-PN-RG were attributed to the expanded V and the decreased or unvaried CL. Combination of PN, RG or PN-RG delayed the absorption rate and extent of wilforlide in the intestine of rats. Weighted with the CL, the dose of wilforlide in rats was also reduced by PN, RG and PN-RG. The lower C_max_, more prolonged T_max_ and extended t_1/2_ demonstrated more safety plasma concentration and broader therapeutic period of wilforlide in rats with TW-PN, TW-RG and TW-PN-RG (Figure 4 and Table 5).

## 3. Discussion

Triptolide is the pivotal component of TW described as a double-edged sword. On one hand, triptolide acts as the immunosuppressant, anti-inflammatory medicine, and anti-cancer agent clinically [21,22]. On the other hand, triptolide results in hepatotoxicity and nephrotoxicity accompanied with the routine application [10]. Administration of triptolide with intraperitoneal injection or oral gavage induces morphologically pathological lesions involving hepatocellular hydropic degeneration, vacuolization, nuclei pyknosis, necrosis, and markedly abnormal serum biochemical indexes such as AST, ALT, and lactate dehydrogenase (LDH) in rodent. The promotion of oxidative stress, apoptosis, and autophagy is the basic cellular behavior following triptolide exposure. Emergency evidences of action of triptolide on liver and kidney were provided via combination of proteomics analysis and targeted metabonomics assay of fatty acids, which explore several pathways such as acute-phase response signaling, the antigen presentation pathway, farnesoid X receptor/retinoid X receptor (FXR/RXR) activation, lipopolysaccharides/interleukin (LPS/IL)-1-mediated inhibition of RXR function, and eukaryotic initiation factor 2 (EIF2) signaling. Meanwhile, the abundance of fatty acids containing more than 17 carbons were decreased in triptolide-treated mice [11,12]. According to the criteria of Roussel Uclaf Causality Assessment Method (RUCAM), the approximate two-fold serum ALP in TW-treated rats compared to blank ones indicated the suspended herb induced liver injury (HILI) [23]. The ratio of ALT/ALP is about 1.5, and *R* ≤ 2 hints the cholestatic liver injury. This contradiction of advantages and disadvantages is mainly depending on the plasma concentration of triptolide. During the period of therapeutic, the prerequisite serum level of triptolide is above the minimum effective concentration. Due to the relatively narrow therapeutic window, the fluctuation of triptolide concentration in the duration of action is beyond the minimum toxic concentration for the sensitive organs such as liver and kidney. Simply decreasing the dosage of triptolide or TW reduces both the therapeutic and toxic action.

Administration of triptolide alone or TW extract with triptolide shows a different pharmacokinetic process. The investigation of oral gavage of triptolide in rats found that the T_max_ of triptolide is 0.17 h and the t_1/2_ is 0.42 h. While following the administration of TW in our study, the T_max_ and t_1/2_ of triptolide is 2.21 h and 7.7 h [24]. The prolonged T_max_ indicates that the faster absorption of triptolide was obviously delayed by another component of TW which might attribute to the competitive transport. The CL of triptolide was reduced from 6.67 to 1.05 L/h/kg responsible for longer retention time and t_1/2_. The C_max_ closely associated with the action of therapeutic and toxic is 293 μg/L, in rat with pure triptolide of 1 mg/kg. In the present study, the C_max_ of triptolide in the rat with TW is only 2.21 μg/L, the equivalent dose of which is about 0.08 mg/kg triptolide. Then administration of TW avoids the rushing elevation of triptolide to a higher serum level in a shorter period. The slow and smooth elevation of triptolide with administration of TW contributes to more safety and longer action span, which is positively beneficial to the treatment application for chronic and complex diseases. 

At present, there are several primary approaches to reinforce the action and weaken the toxicity of triptolide. One way is to reform the formulation with the emerging dosage form. The polymeric micelle, a novel drug delivery carrier, increases the lethal dose, 50% (LD_50_) of triptolide administered intravenously from 0.83 mg/kg to 1.06 mg/kg and significantly decreases the subacute toxicity of triptolide on the liver, kidney, testis, and spleen [25]. Otherwise, a triptolide-loaded liposome hydrogel patch was developed which carries more dose of triptolide to the blood in a similar C_max_ with triptolide singly [26]. Moreover, solid lipid nanoparticle delivery system provides appreciated toxicokinetic process of reduced C_max_ and increased AUC in rats [14]. Another way is to combine with other chemicals having mimic actions which archives these expected treatment effects in a relatively lower dose. The combination therapy of sorafenib and triptolide on hepatocellular carcinoma affords greater efficacy than single-agent treatments in decreasing cell viability, inducing caspase-3, caspase-7 activity, and inhibiting NF-κB function [17]. Others like inducer of cytochrome P450 (CYP) enzyme, CYP3A, and chemical structure transformation have been adopted to regulate the pharmacokinetics process or the therapeutic index of triptolide [27,28,29]. The application of TW and compatibility with other herbs in traditional Chinese medicine reduce the mass of triptolide in a prescription based on the support of other chemicals with similar structure and various target-specific compound from the fellow herbs, and modulate the pharmacokinetic characteristics spontaneously. The data of our study show that both PN and RG change the trip of triptolide in rat responsible for the reduction of toxic effect of TW on the liver and kidney. The similar pharmacokinetic profiling of wilforlide was demonstrated. Undoubtedly, the role of PN and RG are not limited in regulating the amount of triptolide in the body according to the compatible principle.

However, there are still some obscure problems that have not explained in this investigation and needed further rigorous study. (1) Besides the alteration of pharmacokinetic process of triptolide and wilforlide A, whether PN and RG block the toxic action of TW on liver and kidney, and the possible molecular mechanisms are indistinct yet. (2) The exact ingredients of PN and RG that perform interaction with triptolide, wilforlide A, or other chemicals of TW should be further explored. (3) The tissue distribution of triptolide and wilforlide A in the kidney and liver should be analyzed, accompanied with the further toxic action assay. (4) Proteomics, transcriptomics, and metabonomics assays are required to unravel the complicated network underlying the pharmacologic and toxic actions of TW with PN and/or RG. (5) How to popularize the application of this compatible formulas beyond the limited application within some communities? Randomized controlled trails (RCTs), the golden standard of clinical trial avoiding ambiguity and bias, should finally be adopted to test the efficacy or effectiveness of that compatible formula aforementioned.

## 4. Materials and Methods

### 4.1. Chemicals and Reagents

**Figure 5:**
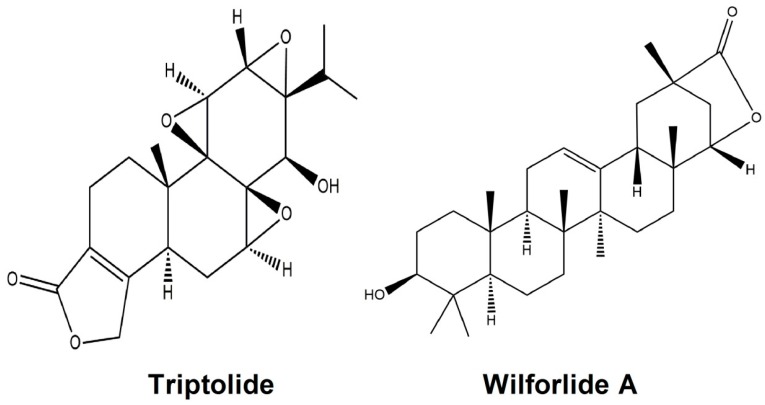
Chemical structure of triptolide and wilforlide A.

TW was purchased from Xichang Materials Company of Sichuan (Xichang, China), while PN and RG was collected at Bozhou Medicine Company of Anhui (Bozhou, China). All these herbal medicinals were identified by Qi’nan Wu (Department of Pharmacognosy, College of Pharmacy, Nanjing University of Chinese Medicine, Nanjing, China) according to the standard of Chinese pharmacopoeia (2010). Triptolide, wilforlide A (Figure 5), and fenofibrate were from the National Institute for the Control of Pharmaceutical and Biological products (Beijing, China). The kits of T-BIL, ALT, AST, ALP, Bun, and SCr were obtained from Nanjing Jiancheng Bioengineering Institute (Nanjing, China). Acetonitrile and methanol used for UPLC were of chromatographic grade (Merck, Darmstadt, Germany). All of other reagents were of analytical grade (Sino Pharm Chemical Reagent Co., Ltd., Shanghai, China). Milli-Q water (Millipore, Bedford, MA, USA) was used throughout the study.

### 4.2. Preparation of the Extracts of Herbal Materials

Herbal materials of TW (1200 g), TW-PN (1200 g + 360 g), TW-RG (1200 g + 1500 g), TW-PN-RG (1200 g + 360 g + 1500 g), crushed respectively to pieces were soaked in cold water (1:11, *w*/*v*) for 1.5 h and then extracted with boiling water for 1 h. The residue was collected and extracted again in the boiling fresh water (1:7, *w*/*v*) for 1 h. The decoction merged and filtrated through gauze was evaporated to 300 mL by rotary evaporator systems (RV8, IKA, Staufen, Germany) under condition of vacuum.

### 4.3. Animal Experiment

Male Sprague-Dawley rats (250 ± 10 g) from Zhejiang Province Laboratory Animal Center (Hangzhou China) were kept in a breeding room (temperature, 25 ± 1 °C; humidity, 65 ± 5%; 12 h light/dark cycle) for one week before the onset of experiment. The study was carried out strictly accordance with the recommendations in the Guide for the Care and Use of Laboratory Animals of the National Institutes of Health. The study protocol and the total number of rat were approved by the Animal Care and Use Committee of Nanjing University of Chinese Medicine strictly following the guideline of for the Care and Use of Laboratory Animals published by the US National Institutes of Health (NIH Publication No. 85-23, revised 20 May 1985).

To assay the damage of these receipts on liver and kidney, thirty rats were randomly and evenly divided into five groups, blank, TW, TW-PN, TW-RG, and TW-PN-RG. The rats were administrated intragastrically with corresponding extract solution at a dosage of 10 mL/kg for seven days. Blank rats were given normal saline with similar volume. At the day 3 and 7, blood samples (1 mL) were withdrawn from the orbit venous plexus of rats 1.5 h after the administration and centrifuged at 3000 rpm for 10 min to harvest the serum, in which the levels of T-BIL, ALT, AST, ALP, Bun, and SCr were determined with commercial kits according to the manufacturer’s instruction . The rats were fasted and free access to water for 12 h. Meanwhile, the rats were sacrificed at day 7, and the liver and kidney tissue were taken, ashed with saline solution and fixed in 10% formaldehyde solution for hematoxylin and eosin stain performed in the pathology laboratory of Nanjing Medical University for histopathological examination.

To analyze the pharmacokinetics properties of triptolide and wilforlide A, twenty-four rats were randomly and evenly divided into four groups, TW, TW-PN, TW-RG, and TW-PN-RG. Following the singly oral administration of medicinal materials extract (10 mL/kg), blood samples (0.5 mL) were serially taken from the orbit venous plexus of rats to heparinized centrifuge tubes at 0.083, 0.166, 0.25, 0.5, 0.75, 1, 1.5, 2, 3, 4, 6, 8, 12, and 24 h. The rats were replenished with sterile saline 10 mL/kg subcutaneously at 2, 4 h. To collect plasma, blood were centrifuged at 12,000 rpm at 4 °C for 10 min, and aliquot of supernatant (200 μL) was transferred into another tube and stored at −80 °C for analysis of triptolide and wilforlide A within 1 week.

### 4.4. Sample Preparation

The harvested plasma 200 μL was mixed with fenofibrate 10 μL (500 ng/mL) as the internal standard and methanol 500 μL. After vortex for 2 min, the mixture was centrifuged at 12,000 rpm for 10 min, and the supernatant was transferred into another tube. After condensing with vacuum centrifugal concentrators (CentriVap, Thermo Fisher Scientific, Waltham, MA, USA) at 4 °C, the precipitate was redissolved in 100 μL acetonitrile. Finally, the solution was centrifuged again at 12,000 rpm for 10 min and 20 μL supernatant was adopted to analyze by UPLC-MS/MS system.

### 4.5. UPLC-MS/MS Analysis

The analysis of triptolide and wilforlide A was performed via LC-30A ultra-high performance liquid chromatograph (UPLC, Shimadzu, Kyoto, Japan) and Triple Quad 4500 mass spectrometers system with API 4000 platform (AB SCIEX, Framingham, MA, USA). The elution of triptolide, wilforlide A and fenofibrate was carried out in a Zorbax Eclipse Plus C18 column (150 mm × 2.1 mm inner diameter, 3.5 μm particle size, Agilent, Santa Clara, CA, USA) with gradient elution program was as follows: 50% A from 0 to 1 min, 50–80% A from 1 to 5 min, 80% A from 5 to 7 min, 80–98% A from 7 to 15 min, 98% A from 15 to 15.5 min, 98–50% A from 15.5 to 16 min, and 50% A 20.5 min, in which mobile phrase A consisted of acetonitrile and mobile phrase B 0.1% acetic acid (*v*/*v*) in water. The sampling volume was 2 μL and the flow rate of mobile phrase (A + B) was 0.2 mL/min. The column temperature was kept at 30 °C constantly. The whole system was maintained as the initial state for the last one minute.

MS/MS detection was performed using an electrospray ionization source operating in a positive ion mode. Multiple reaction monitoring (MRM) was selected for detection of triptolide, wilforlide A and internal standard with a dwell time of 75 ms. The tune parameters used for data acquisition were: the ion source temperature of 500 °C; a spray voltage of 5000 V; curtain gas of 15 pounds per square inch (psi); nebulizer gas of 40 psi; heating gas of 55 psi; collision activation dissociation (CAD) gas value of 4 psi. Nitrogen (99.995% purity) was used as the desolvation and collision gas. The MRM acquisition method was run in unit resolution (0.7 amu) in both Q1 and Q3. The *m*/*z* of triptolide and wilforlide A was 451.2/201.1 and 455.3/409.2. The corresponding declustering potential was 120 V and 48 V and the collision energy was 37 eV and 25 eV. The representative chromatograms are shown in Figure 3.

### 4.6. Method Validation

The methods for quantitative analysis of triptolide and wilforlide A in plasma samples were validated according to the requirement of biopharmaceutical analysis, which was examined for specificity, linearity, precision, extraction recovery, and stability under the UPLC analytical conditions.

The specificity was evaluated by comparing rat blank plasma, rat plasma spiked with fenofibrate, triptolide, and wilforlide A and rat plasma sample after administration of TW.

The precision was determined from inter-day and intra-day using five sets of plasma quality control (QC) samples and was expressed by the relative standard deviation (RSD%), which was estimated as follows: RSD (%) = (standard deviation (SD)/the observed concentrations of replicate analyses of QC samples (Cobs)) × 100.

The stability of the method was evaluated by analyzing plasma QC samples which thawing at constant temperature water bath (37 °C) after stored at −85 °C for 7 days. The freezing and thawing process was repeated for three times.

The extraction recoveries were determined by calculating the ratio of triptolide and wilforlide A detected in plasma QC samples against those initially added in blank plasma.

### 4.7. Pharmacokinetic Parameters Analysis

Those pharmacokinetic parameters was estimated by the DAS 2.1 software package from Mathematical Pharmacology Professional Committee of China (Shanghai, China), which include maximum concentration (C_max_), time to reach C_max_ (T_max_), half-life (t_1/2_), apparent volume of distribution (V_d_/F), clearance (CL/F), area under plasma concentration-time curve from zero to the last designated time (AUC_0–t_), as well as from zero to infinity (AUC_0–∞_), by two-compartmental model methods. Statistical analysis was performed by Student’s *t*-test and one-way ANOVA with *post hoc* tests via the SPSS program (version 18.0, IBM Corp., Chicago, IL, USA).

### 4.8. Statistical Analysis

The data were analyzed via one-way ANOVA and expressed as means ± standard deviation. Following the statistically significance (*p* < 0.05) indicated in the ANOVA results, the Newman-Keuls *post hoc* analysis was adopted for pair wise multiple comparisons. ^##^ *p* < 0.01, compared with blank; ** *p* < 0.01, compared with TW.

## 5. Conclusions

In the present investigation, the formula composed of TW, PN and RG shows less toxic action on liver and kidney than that of TW absence of PN and RG. The synergism of PN and RG in decreasing the C_max_ and AUC of triptolide from TW in rats contributes to the detoxication, which might be involved in the absorption process and tissue distribution of triptolide and wilforlide A. The change of intracorporal process of activity components initiated by PN and RG supported one of the most important mechanisms underlying the rational and feasible application of compatibility with TW based on the theory of traditional Chinese medicine. 

## Figures and Tables

**Table 1:** Analytical precision of triptolide and wilforlide A in perfusate samples (inter-day *n* = 5; intra-day *n* = 5).

Metrics Component	Low Concentration		Middle Concentration		High Concentration	
	Intra-Day	Inter-Day	Intra-Day	Inter-Day	Intra-Day	Inter-Day
triptolide	2.54%	4.67%	2.49%	5.23%	2.61%	5.56%
wilforlide A	4.04%	5.06%	3.92%	5.74%	4.15%	6.32%

**Table 2:** The levels of T-BIL, ALT, AST and ALP in rat serum (*n* = 6, mean ± SD).

Groups		T-BIL (μmol/L)	ALT (U/L)	AST (U/L)	ALP (King’s Unit/100 mL)
Blank	3 days	24.28 ± 2.81	45.67 ± 2.51	108. 36 ± 8.65	22.36 ± 1.35
	7 days	25.14 ± 2.22	44.85 ± 2.37	107.88 ± 8.16	22.83 ± 1.29
TW	3 days	46.32 ± 1.89 ^##^	55.62 ± 2.09 ^##^	131.49 ± 6.83 ^##^	35.62 ± 1.43 ^##^
	7 days	49.58 ± 1.53 ^##^	61.63 ± 2.25 ^##^	153.74 ± 7.94 ^##^	40.73 ± 1.42 ^##^
TW-PN	3 days	30.38 ± 2.06 **	45.88 ± 2.07 **	115.65 ± 7.59 **	28.27 ± 1.95 **
	7 days	35.14 ± 2.03 **	50.85 ± 2.04 **	127.82 ± 8.16 **	34.34 ± 1.38 **
TW-RG	3 days	33.45 ± 1.94 **	47.55 ± 1.88 **	128.65 ± 7.76	31.43 ± 1.75 **
	7 days	37.46 ± 2.62 **	52.97 ± 2.35 **	136.56 ± 8.35 **	38.92 ± 1.62
TW-PN-RG	3 days	26.69 ± 2.31 **	46.65 ± 2.74 **	112.49 ± 7.74 **	26.55 ± 1.53 **
	7 days	31.13 ± 2.47 **	49.88 ± 1.96 **	116.55 ± 8.64 **	30.15 ± 1.27 **

T-BIL, total bilirubin; ALT, alanine amino transferase; AST, aspartate amino transferase; ALP, alkaline phosphatase; TW, *Tripterygium wilfordii*; PN, *Panax notoginseng*; RG, *Rehmannia glutinosa*. ^##^ *p* < 0.01, compared with blank; ** *p* < 0.01, compared with TW of corresponding time points.

**Table 3:** The levels of BUN and SCr in rat serum (*n* = 6, mean ± SD).

Groups		BUN (mmol/L)	SCr (μmol/L)
Blank	3 days	15.28 ± 2.81	20.12 ± 1.51
	7 days	18.67 ± 2.68	20.33 ± 1.77
TW	3 days	49.58 ± 1.53 ^##^	36.46 ± 1.79 ^##^
	7 days	60.88 ± 2.21 ^##^	49.82 ± 2.35 ^##^
TW-PN	3 days	35.14 ± 2.03 **	30.68 ± 2.11 **
	7 days	46.49 ± 2.53 **	33.54 ± 2.46 **
TW-RG	3 days	37.46 ± 2.62 **	34.51 ± 2.27
	7 days	50.33 ± 1.83 **	38.57 ± 2.55 **
TW-PN-RG	3 days	31.13 ± 2.47 **	28.39 ± 2.08 **
	7 days	35.67 ± 1.72 **	30.48 ± 1.98 **

BUN, blood urea nitrogen; SCr, serum creatinine. ^##^ *p* < 0.01, compared with blank; ** *p* < 0.01, compared with TW of corresponding time points.

**Table 4:** The pharmacokinetics parameters of triptolide in rats (*n* = 6, mean ± SD).

	TW	TW-PN	TW-RG	TW-PN-RG
AUC_(0–t)_/μg·h·L^−1^	17.45 ± 1.35	15.43 ± 0.27	16.10 ± 0.16	14.84 ± 0.29
AUC_(0–∞)_/μg·h·L^−1^	18.93 ± 1.17	16.71 ± 0.18	17.25 ± 0.33	17.10 ± 0.18
t_1/2_/h	7.07 ± 0.87	7.11 ± 0.33	6.69 ± 0.25	9.31 ± 0.19
T_max_/h	2.21 ± 0.12	2.30 ± 0.16	2.65 ± 0.13	2.72 ± 0.22
V_d_/F/L·kg^−1^	10.78 ± 1.25	12.28 ± 1.32	11.21 ± 1.34	15.72 ± 1.21
CL/F/L·h^−1^·kg^−1^	1.05 ± 0.26	1.19 ± 0.16	1.15 ± 0.19	1.16 ± 0.39
C_max_/μg·L^−1^	2.21 ± 0.52	1.81 ± 0.69	2.00 ± 0.35	1.79 ± 0.13

AUC, area under plasma concentration-time curve; t_1/2_, half-life; T_max_, time to reach C_max_; V_d_, apparent volume of distribution; CL, clearance; C_max_, reduced maximum concentration.

**Table 5:** The pharmacokinetics parameters of wilforlide A in rats (*n* = 6, mean ± SD).

	TW	TW-PN	TW-RG	TW-PN-RG
AUC_(0–t)_/μg·h·L^−1^	3.93 ± 0.35	2.84 ± 0.59	3.09 ± 0.32	2.54 ± 0.21
AUC_(0–∞)_/μg·h·L^−1^	15.58 ± 0.24	12.82 ± 1.55	7.50 ± 0.61	5.64 ± 0.48
t_1/2_/h	14.12 ± 0.84	86.96 ± 2.39	32.34 ± 1.33	25.86 ± 1.58
T_max_/h	1.52 ± 0.12	2.21 ± 0.69	4.13 ± 0.25	2.52 ± 0.51
V_d_/F/L·kg^−1^	73.04 ± 2.53	169.30 ± 5.68	124.43 ± 3.98	132.17 ± 3.76
CL/F/L·h^−1^·kg^−1^	3.58 ± 0.46	1.34 ± 0.29	2.66 ± 0.36	3.54 ± 0.41
C_max_/μg·L^−1^	0.24 ± 0.043	0.19 ± 0.064	0.21 ± 0.062	0.17 ± 0.051

## Supplementary Materials

Supplementary File 1

## Abbreviations

TW	*Tripterygium wilfordii*
SLN	solid lipid nanoparticles
PN	*Panax notoginseng*
RG	*Rehmannia glutinosa*
UPLC-MS/MS	Ultra-high performance liquid chromatography-tandem mass spectrometry
T-BIL	kits of total bilirubin
ALT	alanine aminotransferase
AST	aspartate aminotransferase
ALP	alkaline phosphatase
Bun	blood urea nitrogen
SCr	serum creatinine
T_max_	time to reach C_max_
C_max_	reduced maximum concentration
AUC	area under plasma concentration-time curve
t_1/2_	half-life
V_d_	apparent volume of distribution
NF-κB	nuclear factor kappa-light-chain-enhancer of activated B cells
UPLC	Ultra-high performance liquid chromatography
RSD	relative standard deviation
CL	clearance
LDH	lactate dehydrogenase
FXR	farnesoid X receptor
RXR	retinoid X receptor
LPS	lipopolysaccharides
IL	interleukin
EIF2	eukaryotic initiation factor 2
LD_50_	Lethal Dose, 50%
psi	pounds per square inch
amu	atomic mass units
QC	quality control
