# Supplementary figures and images for: Compatibility with Panax notoginseng and Rehmannia glutinosa Alleviates the Hepatotoxicity and Nephrotoxicity of Tripterygium wilfordii via Modulating the Pharmacokinetics of Triptolide
Source: Int J Mol Sci. 2018 Jan 19;19(1):305. doi: 10.3390/ijms19010305 (PMC5796250; doi:10.3390/ijms19010305)

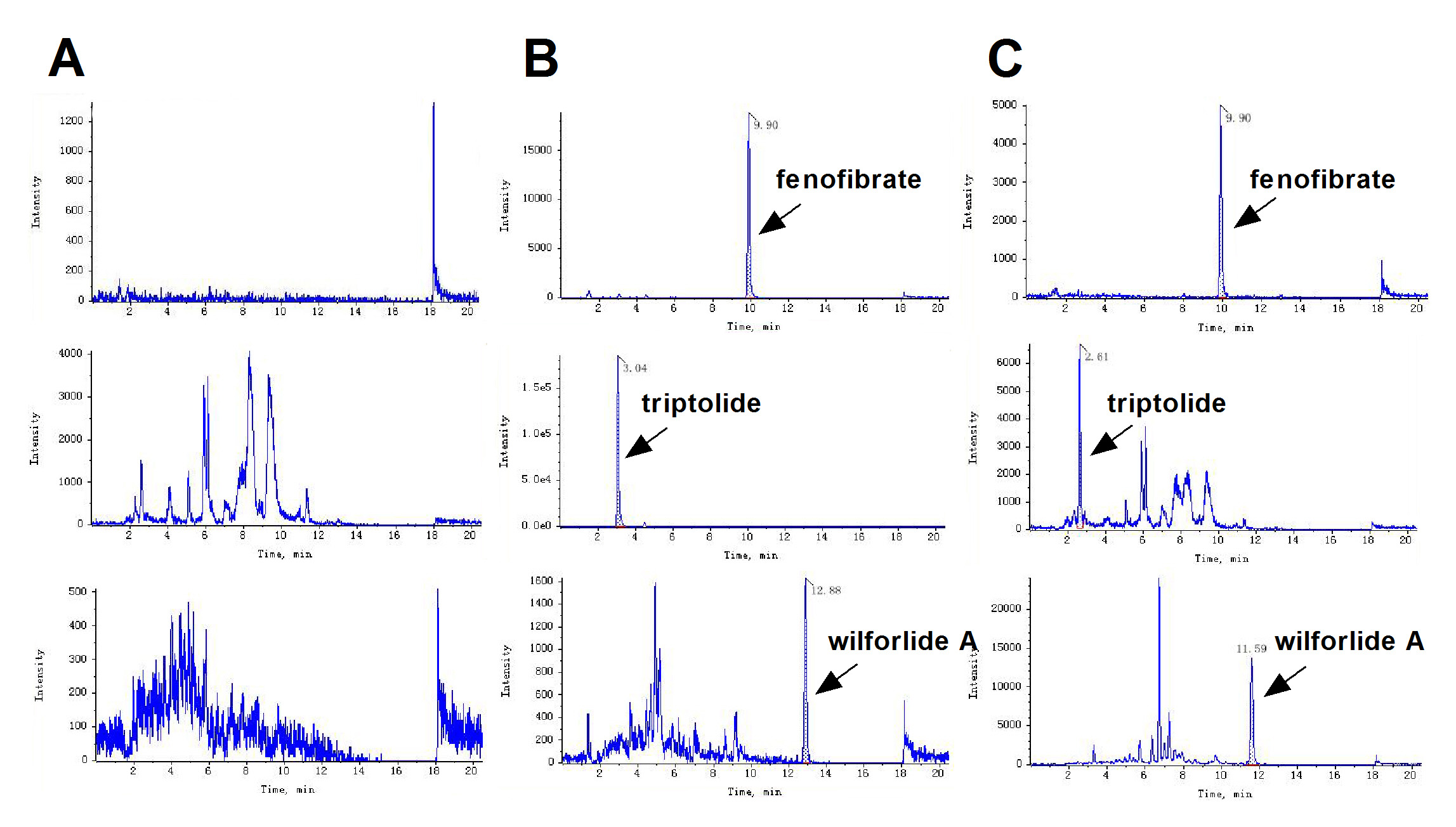

Supplement: Supplementary File 1 [file ijms-19-00305-s001.zip › Supplementary Materials/Figure S1.jpg]

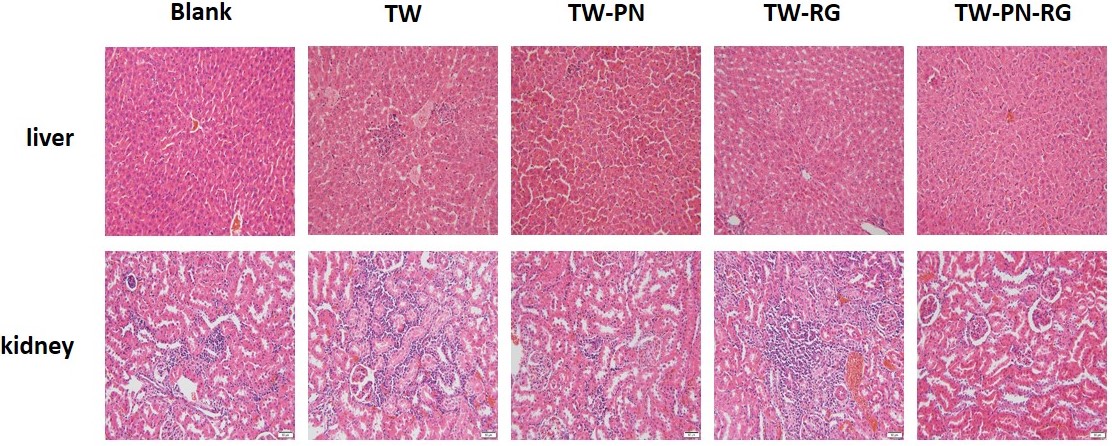

Supplement: Supplementary File 1 [file ijms-19-00305-s001.zip › Supplementary Materials/Figure S2.jpg]

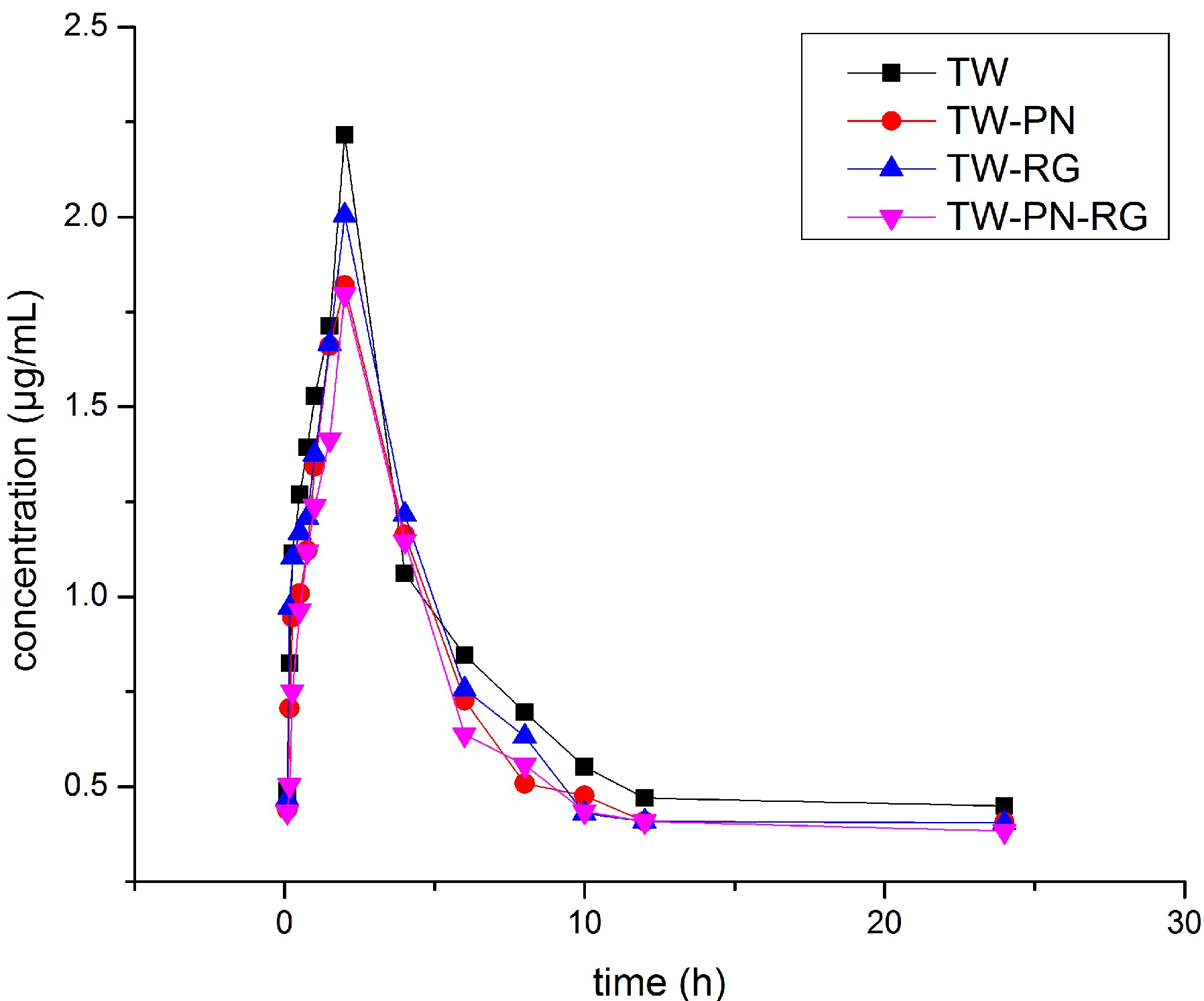

Supplement: Supplementary File 1 [file ijms-19-00305-s001.zip › Supplementary Materials/Figure S3.jpg]

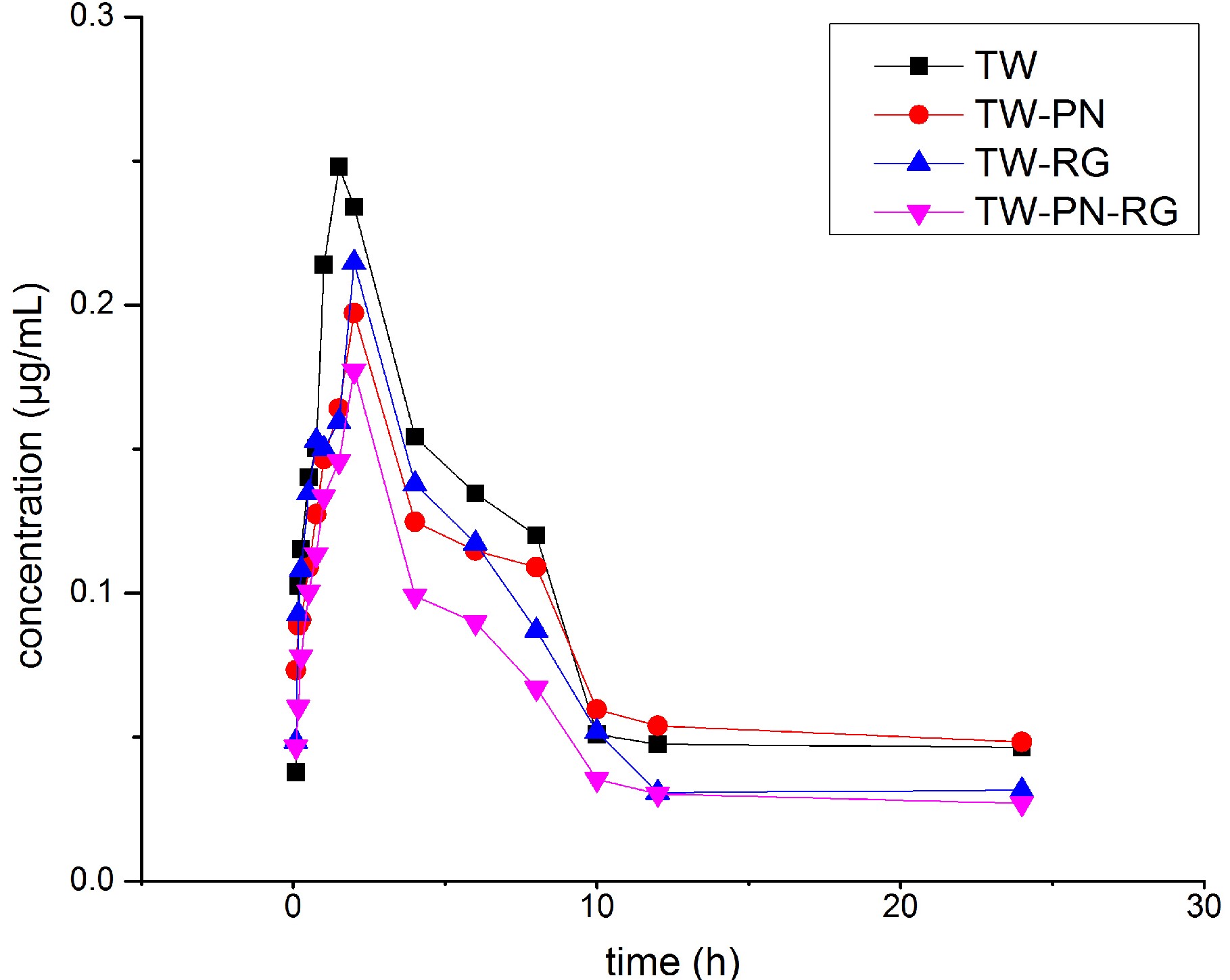

Supplement: Supplementary File 1 [file ijms-19-00305-s001.zip › Supplementary Materials/Figure S4.jpg]

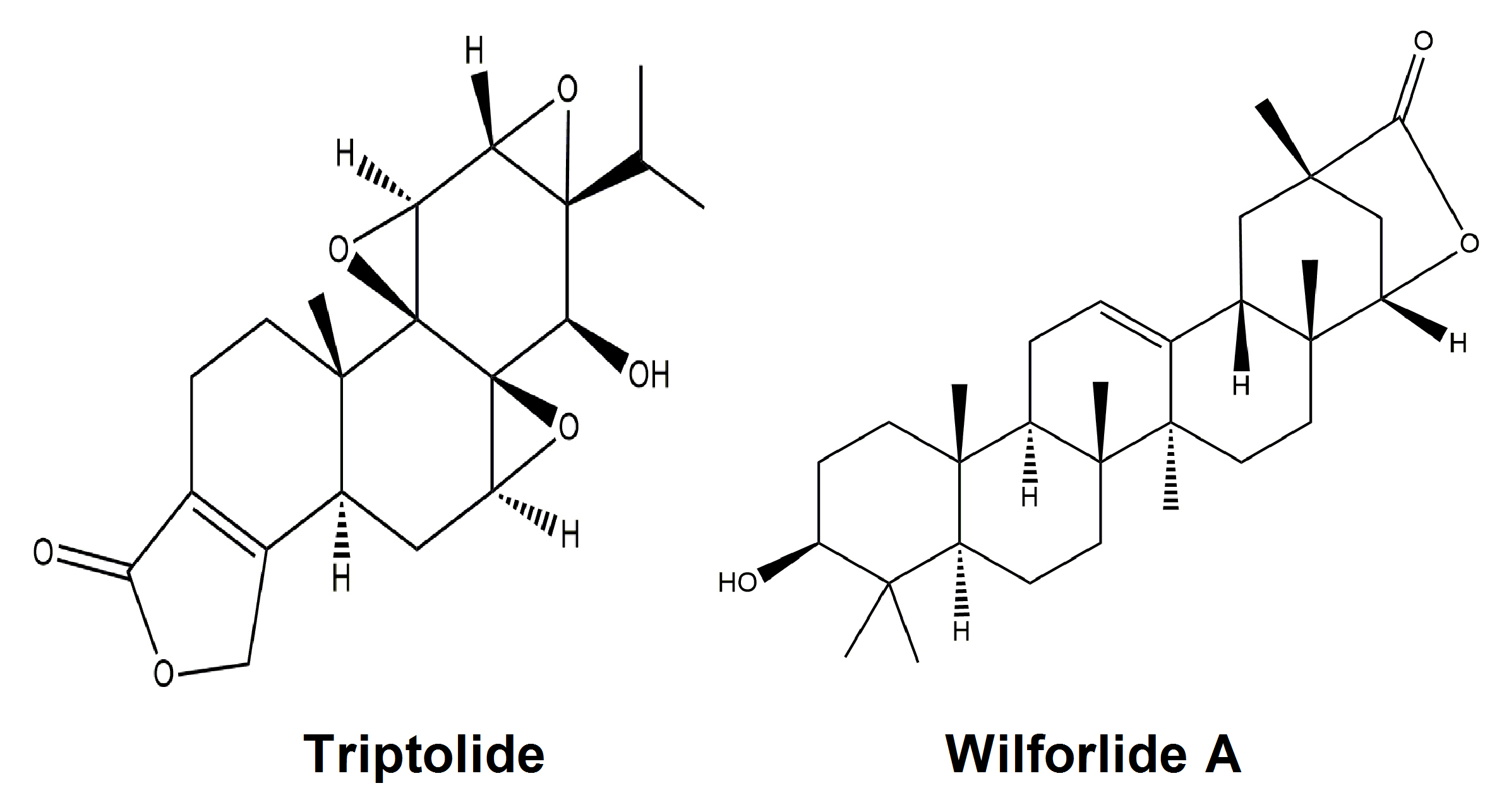

Supplement: Supplementary File 1 [file ijms-19-00305-s001.zip › Supplementary Materials/Figure S5.jpg]
