# Supplementary material for: Compatibility with Panax notoginseng and Rehmannia glutinosa Alleviates the Hepatotoxicity and Nephrotoxicity of Tripterygium wilfordii via Modulating the Pharmacokinetics of Triptolide
Source: Int J Mol Sci. 2018 Jan 19;19(1):305. doi: 10.3390/ijms19010305 (PMC5796250; doi:10.3390/ijms19010305)
Supplement: Supplementary File 1 [file ijms-19-00305-s001.zip › Supplementary Materials/Tables.doc]

Table 1. Analytical precision of triptolide and wilforlide A in perfusate samples (inter-day *n=5*; intra-day *n=5*)

| Metrics component | Low concentration | | Middle concentration | | High concentration | |
| --- | --- | --- | --- | --- | --- | --- |
|  | intra-day | inter-day | intra-day | inter-day | intra-day | inter-day |
| triptolide | 2.54% | 4.67% | 2.49% | 5.23% | 2.61% | 5.56% |
| wilforlide A | 4.04% | 5.06% | 3.92% | 5.74% | 4.15% | 6.32% |

Table 2. The levels of T-BIL, ALT, AST and ALP in rat serum (n=6, $\overline{x}\pm\text{sd}$)

| Groups | | T-BIL(μmol/L) | ALT(U/L) | AST(U/L) | ALP (King’s unit/100 mL) |
| --- | --- | --- | --- | --- | --- |
| Blank | 3d | 24.28 ±2.81 | 45.67 ±2.51 | 108. 36 ± 8.65 | 22.36 ± 1.35 |
|  | 7d | 25.14 ±2.22 | 44.85 ±2.37 | 107.88 ± 8.16 | 22.83 ±1.29 |
| TW | 3d | 46.32 ±1.89^##^ | 55.62 ±2.09^##^ | 131.49 ±6.83^##^ | 35.62 ±1.43^##^ |
|  | 7d | 49.58 ±1.53^##^ | 61.63 ±2.25^##^ | 153.74 ± 7.94^##^ | 40.73 ± 1.42^##^ |
| TW-PN | 3d | 30.38±2.06** | 45.88±2.07** | 115.65±7.59** | 28.27±1.95** |
|  | 7d | 35.14±2.03** | 50.85±2.04** | 127.82±8.16** | 34.34±1.38** |
| TW-RG | 3d | 33.45±1.94** | 47.55±1.88** | 128.65±7.76 | 31.43±1.75** |
|  | 7d | 37.46±2.62** | 52.97±2.35** | 136.56±8.35** | 38.92±1.62 |
| TW-PN-RG | 3d | 26.69±2.31** | 46.65±2.74** | 112.49±7.74** | 26.55±1.53** |
|  | 7d | 31.13±2.47** | 49.88±1.96** | 116.55±8.64** | 30.15±1.27** |

\## *P<*0.01, compared with blank; ** *P<*0.01, compared with TW of corresponding time points

Table 3. The levels of BUN and SCr in rat serum (n=6, $\overline{x}\pm\text{sd}$)

| Groups | | BUN(mmol/L) | SCr(μmol/L) |
| --- | --- | --- | --- |
| Blank | 3d | 15.28 ±2.81 | 20.12 ± 1.51 |
|  | 7d | 18.67 ±2.68 | 20.33 ±1.77 |
| TW | 3d | 49.58 ±1.53^##^ | 36.46 ±1.79^##^ |
|  | 7d | 60.88 ±2.21^##^ | 49.82 ± 2.35^##^ |
| TW-PN | 3d | 35.14 ±2.03** | 30.68 ±2.11** |
|  | 7d | 46.49 ±2.53** | 33.54 ±2.46** |
| TW-RG | 3d | 37.46 ±2.62** | 34.51 ±2.27 |
|  | 7d | 50.33 ±1.83** | 38.57 ±2.55** |
| TW-PN-RG | 3d | 31.13 ±2.47** | 28.39 ±2.08** |
|  | 7d | 35.67 ±1.72** | 30.48 ±1.98** |

\## *P<*0.01, compared with blank; ** *P<*0.01, compared with TW of corresponding time points

Table 4. The pharmacokinetics parameters of triptolide in rats (n=6, $\overline{x}\pm\text{sd}$)

|  | TW | TW-PN | TW-RG | TW-PN-RG |
| --- | --- | --- | --- | --- |
| AUC_(0-t)_/ μg·h·L^-1^ | 17.45±1.35 | 15.43±0.27 | 16.10±0.16 | 14.84±0.29 |
| AUC_(0-∞)_/ μg·h·L^-1^ | 18.93±1.17 | 16.71±0.18 | 17.25±0.33 | 17.10±0.18 |
| t_1/2_/ h | 7.07±0.87 | 7.11±0.33 | 6.69±0.25 | 9.31±0.19 |
| T_max_/ h | 2.21±0.12 | 2.30±0.16 | 2.65±0.13 | 2.72±0.22 |
| Vd/F/ L·Kg^-1^ | 10.78±1.25 | 12.28±1.32 | 11.21±1.34 | 15.72±1.21 |
| CL/F/ L·h^-1^·Kg^-1^ | 1.05±0.26 | 1.19±0.16 | 1.15±0.19 | 1.16±0.39 |
| C_max_/ ug·L^-1^ | 2.21±0.52 | 1.81±0.69 | 2.00±0.35 | 1.79±0.13 |

Table 5. The pharmacokinetics parameters of wilforlide A in rats (n=6, $\overline{x}\pm\text{sd}$)

|  | TW | TW-PN | TW-RG | TW-PN-RG |
| --- | --- | --- | --- | --- |
| AUC_(0-t)_/ μg·h·L^-1^ | 3.93±0.35 | 2.84±0.59 | 3.09±0.32 | 2.54±0.21 |
| AUC_(0-∞)_/ μg·h·L^-1^ | 15.58±0.24 | 12.82±1.55 | 7.50±0.61 | 5.64±0.48 |
| t_1/2_/ h | 14.12±0.84 | 86.96±2.39 | 32.34±1.33 | 25.86±1.58 |
| T_max_/ h | 1.52±0.12 | 2.21±0.69 | 4.13±0.25 | 2.52±0.51 |
| V/F/ L·Kg^-1^ | 73.04±2.53 | 169.30±5.68 | 124.43±3.98 | 132.17±3.76 |
| CL/F/ L·h^-1^·Kg^-1^ | 3.58±0.46 | 1.34±0.29 | 2.66±0.36 | 3.54±0.41 |
| C_max_/ ug·L^-1^ | 0.24±0.043 | 0.19±0.064 | 0.21±0.062 | 0.17±0.051 |
